# The Challenges of Predicting Drug-Induced QTc Prolongation in Humans

**DOI:** 10.1093/toxsci/kfac013

**Published:** 2022-02-11

**Authors:** Jean-Pierre Valentin, Peter Hoffmann, Catherine Ortemann-Renon, John Koerner, Jennifer Pierson, Gary Gintant, James Willard, Christine Garnett, Matthew Skinner, Hugo M Vargas, Todd Wisialowski, Michael K Pugsley

**Affiliations:** Department of Investigative Toxicology, UCB Biopharma SRL, Braine-l’Alleud B-1420, Belgium; Independent, Beaufort, South Carolina 29901, USA; Sanofi, Translational Medicine and Early Development, Bridgewater, New Jersey 08807, USA; Center for Drug Evaluation and Research, FDA, Silver Spring, Maryland 20993, USA; Health and Environmental Sciences Institute, Washington, District of Columbia 20005, USA; AbbVie, Abbott Park, Illinois 60044, USA; Center for Drug Evaluation and Research, FDA, Silver Spring, Maryland 20993, USA; Center for Drug Evaluation and Research, FDA, Silver Spring, Maryland 20993, USA; Vivonics Preclinical Ltd, Nottingham SK10 4TG, UK; Department of Safety Pharmacology & Animal Research Center, Amgen, Thousand Oaks, California 91320, USA; Department of Safety Pharmacology, Pfizer, Groton, Connecticut 06340, USA; Department of Toxicology, Cytokinetics, South San Francisco, California 94080, USA

**Keywords:** proarrhythmia, regulatory, discordance, hERG, QT prolongation, sensitivity, specificity, pharmacokinetics, Torsades de Pointes (TdP)

## Abstract

The content of this article derives from a Health and Environmental Sciences Institute (HESI) consortium with a focus to improve cardiac safety during drug development. A detailed literature review was conducted to evaluate the concordance between nonclinical repolarization assays and the clinical thorough QT (TQT) study. Food and Drug Administration and HESI developed a joint database of nonclinical and clinical data, and a retrospective analysis of 150 anonymized drug candidates was reviewed to compare the performance of 3 standard nonclinical assays with clinical TQT study findings as well as investigate mechanism(s) potentially responsible for apparent discrepancies identified. The nonclinical assays were functional (*I*_Kr_) current block (Human ether-a-go-go related gene), action potential duration, and corrected QT interval in animals (*in vivo* corrected QT). Although these nonclinical assays demonstrated good specificity for predicting negative clinical QT prolongation, they had relatively poor sensitivity for predicting positive clinical QT prolongation. After review, 28 discordant TQT-positive drugs were identified. This article provides an overview of direct and indirect mechanisms responsible for QT prolongation and theoretical reasons for lack of concordance between clinical TQT studies and nonclinical assays. We examine 6 specific and discordant TQT-positive drugs as case examples. These were derived from the unique HESI/Food and Drug Administration database. We would like to emphasize some reasons for discordant data including, insufficient or inadequate nonclinical data, effects of the drug on other cardiac ion channels, and indirect and/or nonelectrophysiological effects of drugs, including altered heart rate. We also outline best practices that were developed based upon our evaluation.

Drug-induced ventricular arrhythmia, specifically Torsades de Pointes (TdP), remains a serious public health issue in the development of safe pharmaceuticals (Gintant *et al.*, 2016). Evaluating whether a new medication prolongs the QT interval, which is a risk factor for TdP, is critical for safe drug development and is evaluated using both *in vivo* and *in vitro* models during nonclinical drug development. The importance of detecting the liability for QT prolongation has been reinforced by both nonclinical and clinical regulatory guidelines (International Council for Harmonization of Technical Requirements for Pharmaceuticals for Human Use [ICH] S7B) and (ICH E14) respectively (Anon., 2005a,b, 2015). A key challenge for the drug development community and cardiovascular (CV) clinicians is to understand how the findings derived from nonclinical assays predict QT evaluation outcomes in humans.

The Health and Environmental Sciences Institute (HESI) initiated a consortium more than a decade ago that includes representatives from pharmaceutical companies, global regulatory agencies, and key nonclinical and clinical opinion leaders from the scientific and medical research communities (Trepakova *et al.*, 2009). This consortium is called the HESI Pro-Arrhythmia Working Group and was formed with the goal of understanding how well nonclinical safety methods can assess cardiac ventricular repolarization (Hanson *et al.*, 2006) and determining if (or how) cardiac safety can be improved during drug development.

The primary objective of the HESI Pro-Arrhythmia Working Group was to assess the concordance between the results of nonclinical repolarization assays and clinical QT interval prolongation. The first step was a comprehensive literature search, covering the period from 1960 to 2011, to identify both human and nonrodent animal studies that evaluated drug-induced changes in the corrected QT (QTc) interval for the same drug. The search found that, in the last 51 years of published scientific literature, there was a strong qualitative concordance between QT results found in humans and QT results in nonrodent animal studies. Vargas *et al.* (2015) found that 91% of the drugs that prolonged the QT interval in humans also did so in animals and 88% of drugs that did not prolong the QT in humans had no effect on animals (Tables 1 and 2). A lack of concordance did occur for approximately10% of drugs evaluated, and there appeared to be plausible explanations for the underlying disconnect between the human and nonrodent animal QT outcomes (Vargas *et al.*, 2015).

**Table 1.** The Sensitivity, Specificity, and Concordance of Nonclinical Drug Safety *In Vitro* Assays Used to Predict the Clinical QTc Study Outcome

Test System End-Point	Species	Clinical Endpoint^*a*^	No. of Compounds	Exposure Multiple	Sensitivity %	Specificity %	Predictability %	Reference	Data Source
hERG	Human	Clinical QT studies designed to detect a 7–10 ms QTc change	17	2	82	75	79	Wallis (2010)	Proprietary Data
			12	10	90	38	67		
			10	30	90	14	59		
hERG	Human	TQT positive study set as a mean value > 5 ms	39	3	7	100	67	Gintant (2011)	FDA/EMA Approval Packages
			39	10	21	96	69		
			39	30	57	92	64		
			39	100	64	76	72		
hERG	Human	ICH E14 criteria for TQT or based on concentration-response modeling of QTc data for SAD and MAD studies	24	3	15	100	54	Pollard *et al.* (2017)	Proprietary Data
			24	10	38	100	67		
			24	30	69	91	79		
hERG	Human	5 ms evidenced by upper bound of the 95% CI around the mean effect on QTc of 10 ms	41	3	15	89	66	Park *et al.* (2018)	FDA Database
			66	10	33	91	73		
			74	30	50	92	78		
APD (Purkinje fiber)	Dog	Clinical QT studies designed to detect 7–10 ms QTc change	17	2	20	100	50	Wallis (2010)	Proprietary Data
			12	10	33	80	53		
			10	30	100	40	42		
APD (Purkinje fiber, papillary muscle, isolated Heart)	Dog, rabbit, G. Pig, Sheep	5 ms evidenced by upper bound of the 95% CI around the mean effect on QTc of 10 ms	38	3	0	89	58	Park *et al.* (2018)	FDA Database
			52	10	19	94	63		
			54	30	19	91	63		

Abbreviations: APD, action potential duration; CI, confidence interval; PF, Purkinje fiber; PM, papillary muscle; G. Pig, Guinea Pig; ms, millisecond; hERG, human ether-a-go-go related gene; QTc, corrected QT; TQT, thorough QT.

When evaluating the predictability of a model an overall predictive capacity of 65–74% is considered sufficient (denoted in red text); 75–84% is considered good (denoted in gold text) whereas accuracy > 85% is considered excellent (denoted in green text; Genschow *et al.*, 2002).

a According to the Methods section in the reference paper.

**Table 2.** The Sensitivity, Specificity, and Concordance of Nonclinical Drug Safety *In Vivo* Assays Used to Predict the Clinical QTc Study Outcome

Test system end-point	Species	Clinical Endpoint^*a*^	No. of Compounds	Exposure Multiple	Sensitivity %	Specificity %	Predictability %	Reference	Data Source
QTc interval prolongation	Dog	Clinical QT studies designed to detect a 7–10 ms QTc change	17	2	83	86	85	Wallis (2010)	Proprietary Data
			12	10	83	33	58		
			10	30	83	–	50		
QTc interval	Dog	QTc increase, decrease or unchanged as judged by clinical report to be test article related and noted in conclusion	114	3	17	92	86	Ewart *et al.* (2014)	Proprietary Data
			114	10	88	76	78		
			114	30	100	58	67		
QTc interval	Multiple	(see note below)^b^	40	NA	91	88	90	Vargas *et al.* (2015)	PubMed
QTc interval prolongation	Dog	ICHE14 criteria for TQT or based on concentration-response modeling of QTc data for SAD and MAD studies	14	3	63	83	71	Pollard *et al.* (2017)	Proprietary Data
			16	10	73	80	75		
			11	30	89	50	82		
QTc interval prolongation	Dog and monkey	5 ms evidenced by an upper bound of 95% CI around mean effect on QTc of 10 ms	46	3	18	100	80	Park *et al.* (2018)	Proprietary Data
			43	10	15	100	74		
			30	30	57	91	83		

Abbreviations: NA, not applicable; CI, confidence interval; ms, millisecond; TQT, thorough QT; QTc, corrected QT.

a According to the methods section in the reference paper.

b The human QT data were included irrespective of study design, study duration, sample size, specific clinical subject population enrolled, QT data collection method, QT analysis extraction method or QT correction method used because the clinical QT evaluations were performed both before and after the implementation of ICH E14 guideline requirements. The significance of the human and animal QT findings was accepted as reported. When evaluating the predictability of a model an overall predictive capacity of 65–74% is considered sufficient (denoted in red text); 75–84% is considered good (denoted in gold text) whereas accuracy > 85% is considered excellent (denoted in green text; Genschow *et al.*, 2002)

The second step was creating a joint HESI/U.S. Food and Drug Administration (FDA) database of nonclinical and clinical QT data in which a total of 150 drugs, both investigational new drugs (IND) and new drug applications (NDA) submitted to the FDA between 2006 and 2012 were evaluated for concordance between nonclinical repolarization assays and clinical QT/QTc interval prolongation (Park *et al.*, 2018). The nonclinical assays that were evaluated included the rapidly inactivating delayed rectifier potassium ion channel (human ether-a-go-go related gene [hERG]) expressed in mammalian cells, the cardiac action potential duration (APD) recorded from experimental animal preparations (eg, Purkinje fibers) and several animal species (eg, dog, guinea pig, sheep, and rabbit; Table 1), and measurements of the *in vivo* QTc interval in dogs and monkeys (Table 2). Sensitivity increased with higher exposure multiples for the hERG and *in vivo* QTc assays (to 50% and 57% at 30× exposure multiples), whereas remaining consistently low (19% at 30×) for the APD assay. In contrast, specificity of all assays was consistently excellent across all exposure multiples (89–100%). Overall concordance in the database was moderate, and out of 150 drugs evaluated, a total of 47 (31%) were found to be discordant based on a predetermined definition. There were a total of 43 thorough QT (TQT) positive drugs in the database and of those, 28 (65%, overall 19%) were discordant (Park *et al.*, 2018).

These findings formulated the necessary background for the content of this article, in which plausible mechanisms underpinning the lack of concordance observed between nonclinical and clinical QT assays are discussed. This article also reviews discordant TQT positive drugs (*n* = 28) derived from the joint HESI/FDA database (Park *et al.*, 2018). These were drugs in which an increase in QT was demonstrated in a TQT study (or equivalent), but which had no positive signals in any of the nonclinical repolarization assays within 30-fold the clinical reference concentration (CRC). Potential best practices are also discussed in attempt to achieve greater predictivity from nonclinical QT assays used in drug development. This article is not intended as a systematic review of the literature but rather a commentary on the discordant results from the Park *et al.* (2018) paper including supportive information from the literature around possible reasons for discordance.

## PREDICTIVE VALUE OF NONCLINICAL QT EFFECTS FOR CLINICAL QT PROLONGATION

The retrospective analysis of 150 submitted drug applications demonstrated robust specificity (the ability to identify drugs without clinical QTc prolongation) but poor sensitivity (the ability to identify drugs with clinical QTc prolongation) when nonclinical assays were used. The *in vivo* QTc assay provided more robust positive and negative predictive values compared with the *in vitro* assays. Earlier studies confirmed an overall good concordance between nonclinical *in vivo* QTc assays and clinical outcomes using conscious telemetered dogs or monkeys (Ando *et al.*, 2005; Hanson *et al.*, 2006; Pollard *et al.*, 2017; Toyoshima *et al.*, 2005; Vargas *et al.*, 2015; Wallis, 2010).

Hanson *et al.* (2006) qualitatively demonstrated that all 6 QT-positive drugs known to cause TdP in the clinic prolonged the QTc interval in dogs when the Fridericia correction was used. None of the 6 negative drugs showed increases in QTc in this setting. This study also showed that there was a reduction in predictive capacity if the Bazett correction formula was used. It falsely identified 3 drugs (verapamil, diphenhydramine, and propranolol) as weak QTc prolonging drugs with isolated increases in QTc occurring alongside increases in heart rate (Hanson *et al.*, 2006). Similar results in conscious telemetered dogs were described by Toyoshima *et al.* (2005); in which all positive control drugs prolonged the QTc at relevant human plasma concentrations. Most of the drugs tested that lacked effects on QTc in humans showed no effects in other animals, except for nifedipine, which prolonged QTc in dogs. This apparent false-positive effect was likely due to the inappropriate application of the Bazett correction since the drug, at the doses tested, produced a reflex tachycardia and this correction is known to overcorrect QTc at elevated heart rates.

Ando *et al.* (2005) described a study in cynomolgus monkeys using the same experimental protocol at different testing facilities. None of the 6 negative drugs prolonged QTc. Although 70% of the positive drugs prolonged the QTc interval, whereas haloperidol, terfenadine, and thioridazine did not. The reason for a failure to detect QTc interval prolongation by these drugs is not apparent but may be attributable to differences in drug metabolism between species, resulting in lower plasma concentrations, or inappropriate use of the Bazett correction formula for changes in heart rate. It may also be the result of an inadequately powered study (*n* = 4) to appropriately detect an effect (Wallis, 2010).

Wallis (2010) reviewed 19 drugs to determine whether the sensitivity and specificity of the *in vivo* dog assay could predict the outcome of a clinical QT study. At clinical exposure multiples of 2- to 30-fold, the sensitivity of the *in vivo* dog assay was high at 0.83; however, specificity was reduced at > 10-fold clinical exposure multiples. Pollard *et al.* (2017) evaluated clinical QTc data from TQT and Phase I clinical studies and found that, for the 24 drugs tested, 8 out of 13 (62%) drugs that produced clinical increases in QTc in the clinic also prolonged QTc in dogs. Similarly, 10 out of 11 QT-negative drugs that did not increase QTc in the clinic also had no effects on QTc in the dog. A concordance rate of 0.81 was determined for all the drugs examined.

The overall good concordance of nonclinical *in vivo* QTc assays with clinical TQT studies is not surprising because the hearts of larger, nonclinical mammals used in CV safety studies have electrophysiological similarities to human hearts and have a common assay endpoint (QTc interval). This reinforces the importance of the nonclinical *in vivo* assay in drug development. Wallis (2010) discusses that one of the problems of using the current *in vivo* dog study design is that limited pharmacokinetic/pharmacodynamic (PK/PD) evaluations are conducted in dog studies which limit correlation to human drug levels. Thus, there is a need for a modification to the current nonclinical *in vivo* QTc assay study design which is currently being addressed with the ICH E14-S7B Questions and Answer (Q&A) document.

PK/PD studies use mathematical models to predict the effect of drugs on single or multiple study parameters. PK/PD modeling in either TQT studies or phase 1 clinical trials is used to determine if a New Chemical Entity (NCE) prolongs the QTc interval in humans (Anon., 2016; Garnett *et al.*, 2018). This approach is useful for drugs and their major metabolites that prolong the QTc interval by directly inhibiting *I*_Kr_. For a drug that prolongs QTc, the results typify a linear PK/PD relationship over a therapeutic exposure range; however, if a QTc finding is driven by a metabolite effect (vs the parent drug) there is often hysteresis which results in a poor linear fit (Darpo *et al.*, 2015; Johannesen *et al.*, 2014). The PK/PD relationship is used to support dosing regimens and dose adjustments in specific populations as well as to predict the QTc effects of doses that were not directly evaluated in clinical studies.

PK/PD analysis has also been used to clarify ambiguous results in TQT studies that use a by-time analysis, which can be influenced by extreme values at a single time point (Garnett *et al.*, 2008). Besides its obvious use to characterize dose and concentration effects, this approach can also provide insight into discrepancies between nonclinical and clinical results. For example, when the time course of QTc prolongation does not reflect plasma concentrations of the NCE (the hysteresis phenomena), other mechanisms for QTc prolongation should be considered, such as the presence of major metabolites, the ability of the drug to accumulate in tissue, or the inhibition of hERG channel trafficking. Furthermore, in comparison to the more traditional by-time statistical analyses, PK/PD analysis has been shown to significantly increase the statistical power to exclude a 10 ms upper confidence boundary for QTc changes from baseline. PK/PD analyses can also be used to define the exposure-QTc relationship from nonclinical studies.

Although not new, several publications have described results from nonclinical reference compound studies in monkeys and dogs that are comparable to clinical outcomes with drugs such as moxifloxacin (a QTc prolonging drug) and levoceterizine (a non-QTc prolonging drug; Chui *et al.*, 2021; Komatsu *et al.*, 2019; Watson *et al*, 2011). The methods used in both the older and newer studies were similar. Others have used PK/PD modeling from dog telemetry studies to demonstrate that dofetilide will produce hERG inhibition and clinical effects in humans (van der Graaf and Benson, 2011).

It is worth noting that, although the study designs and PK/PD methods may differ and there are conflicting reports on the relative sensitivities between animals and humans (Dubois *et al.*, 2017; Marostica et al., 2016; Parkinson et al., 2013), PK/PD modeling of nonclinical studies has been proposed in the upcoming ICH S7B Q&As to support regulatory decisions regarding the presence or absence of QTc prolongation.

## CASE EXAMPLES FROM THE DATABASE

The HESI/FDA database consists of 150 drugs included in either INDs or NDAs that underwent rigorous clinical studies for potential QT liability. (For a detailed structure of the database, see Park *et al.*, 2018.) It contains information from 142 hERG, 84 APD, and 90 *in vivo* QT assays. This robust dataset allowed researchers to evaluate the nonclinical and clinical concordance of these drugs with cardiac repolarization. Overall, there were a total of 43 TQT-positive drugs, 28 of which were considered discordant TQT-positive (false negatives), where an increase in QT was demonstrated in the TQT study (or equivalent), but which had no positive signals in any of the nonclinical repolarization assays at concentrations within a 30-fold multiple of the CRC (the lowest mean free *C*_max_ showing QT prolongation in the TQT study). Of these 28 drugs, 3 caused clinical QT prolongation (ΔΔQTc) ≥20 ms, 11 caused prolongation between 10 and 20 ms, and 14 caused prolongation < 10 ms.

There was a total of 107 TQT-negative drugs, 8 of which were considered discordant TQT-negative drugs because they had a positive signal in at least 1 of the 3 nonclinical assays (false positives; Tables 1 and 2). In the following sections, we focus on the discordant TQT-positive drugs since the database revealed a high incidence of nonclinical false negatives (65%). Such negative test results inappropriately indicate the lack of a QT prolongation effect when it is present, which could create a potentially serious cardiac safety concern.

### Reasons for Discordance

In this article, we give an overview of potential mechanisms that may result in discordance between clinical and nonclinical repolarization assays. We present some examples of discordant drugs from the HESI/FDA database, focusing on discordant TQT-positive drugs. We also tried to better understand the mechanisms that underpin the lack of concordance observed between clinical TQT and nonclinical repolarization assays. We discuss key points about the conduct of nonclinical repolarization assays, and we highlight best practices that may result in a greater ability to predict QT assessment during drug development.

#### Discordance due to data deficiencies

It is important to note that, of the 28 discordant TQT-positive drugs, only 9 had data from all 3 nonclinical assays available for analysis (hERG, APD, and *in vivo* QT). The remaining 19 drugs had only partial nonclinical datasets.

**Figure 1.**
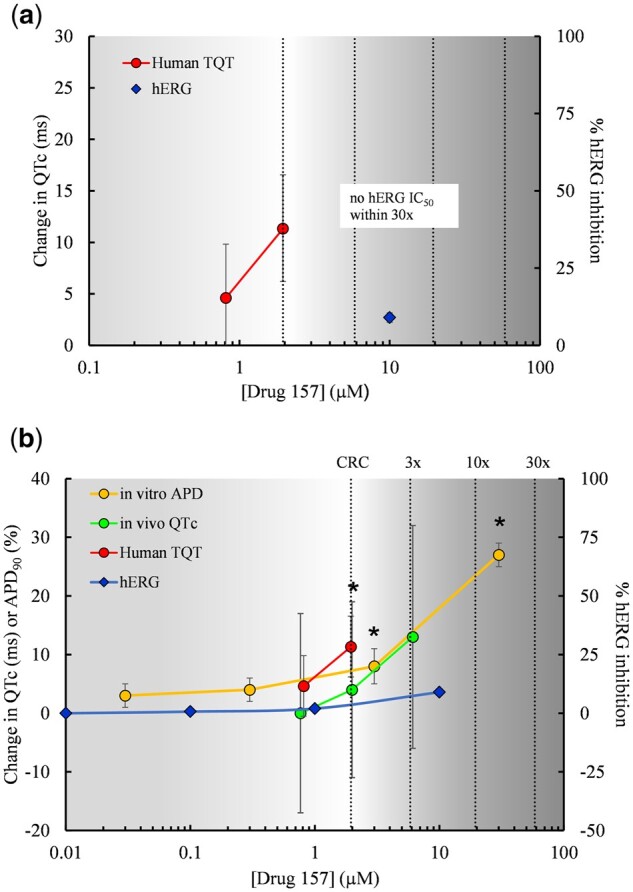
For Drug 157, concentration-response data from the clinical thorough QT (TQT) and nonclinical assays were plotted. The concentration-effect plots show (A) original clinical and nonclinical data for Drug 157 used in the Health and Environmental Sciences Institute/Food Drug Administration analysis by Park *et al.* (2018), and (B) data for Drug 157 with additional nonclinical data supplied by the sponsor. Stars indicate concentrations at which a positive effect occurred in each assay. TQT data are ΔΔ corrected QT (QTc). The *in vitro* action potential duration (APD) data are from a rabbit Purkinje fiber study (APD_90_% change from control), and the *in vivo* QTc data are from an anesthetized dog study (change from control). The addition of supplementary nonclinical data renders Drug 157 concordant with the human TQT result. The vertical clinical reference concentration (CRC) line indicates the lowest *C*_max, free_ showing QT prolongation. The additional vertical lines indicate multiples from the CRC. Data are plasma concentrations of unbound drug.

Figure 1A highlights the nonclinical data for TQT-positive Drug 157, for which only a single point/concentration from the hERG assay was included for FDA review. There were no data from either the APD or *in vivo* QT assays. A 9% inhibition of hERG current was observed at a 10 µM concentration of Drug 157, which is within 10-fold of the CRC defined as the lowest *C*_max, free_ showing QT prolongation for TQT-positive drugs. The data interpretation for Drug 157 was that there was a lack of concordance between the TQT study and the hERG assay (a false negative), since no hERG IC_50_ (half maximal inhibitory concentration) was achieved within 30-fold. However, it is worth noting that the hERG assay for this drug was conducted at a relatively high concentration of K^+^ in the external bath solution (10 vs 4 mM under standard conditions), which can affect IC_50_ estimations and may have impacted the assay results.

Further information gathered from the sponsor after the HESI analysis revealed that Drug 157 was negative in an anesthetized dog *in vivo* QTc study at free plasma concentrations up to 6 µM; however, the data are potentially confounded by the fact that the study was conducted using pentobarbital-anesthetized dogs and the use of this anesthetic in this species results in elevated heart rates that create unfavorable conditions for detecting drug-induced QT prolongation. Drug 157 was positive in an *in vitro* APD assay with significant increases in the APD_90_ observed at concentrations ≥ 3 µM (> 1- to 2-fold the CRC) and higher (Figure 1B). Since the positive effect in the APD assay occurred within 3-fold of the concentration, which is considered a positive effect in the human TQT study, Drug 157 should be reclassified as concordant. This example shows that a more accurate prediction of drug-induced QT liability is achieved by using an integrated assessment of multiple nonclinical assays rather than the hERG assay alone.

It is likely that, in some cases, the lack of either hERG or *in vivo* QTc data, which can both predict clinical QT effects, may have been the reason for the discordance observed between the nonclinical and clinical data. Half of the 28 discordant drugs reviewed lacked either an *in vivo* QT study or hERG assay data and, of the 9 drugs submitted with the full set of nonclinical data, 3 had *in vivo* QTc data in which the plasma concentrations achieved were < 1-fold the CRC, whereas 2 drugs were tested only at plasma concentrations well above the clinical concentration (> 1000-fold the CRC). Predictivity and diagnostic accuracy have both been found to vary with drug plasma concentration multiples, and the *in vivo* QTc assay performs best within a 30-fold multiple from the CRC. It is therefore possible that nonclinical testing at inappropriate doses, along with the resulting very high (or very low) concentration multiples, may have contributed to the discordance noted in the prior HESI/FDA analysis.

#### Concordance threshold considerations

The threshold for evaluating concordance between nonclinical and clinical outcomes in the HESI/FDA project was set at 30-fold. This was based on previous studies suggesting that a therapeutic window of > 30-fold for *in vitro* and *in vivo* repolarization assays or a safety margin > 30-fold between hERG and IC_50_ and the highest clinical *C*_max_ should be sufficient to ensure lack of significant QT prolongation or potential for TdP in the clinic (Redfern *et al.*, 2003; Webster *et al.*, 2002). During the HESI/FDA analysis, TQT-positive drugs with any positive nonclinical results within 30-fold of the CRC were classified as concordant. Of the discordant TQT-positive drugs in the HESI/FDA database, 15 did have a measured or predicted IC_50_ at concentration multiples >30-fold the CRC (5 within 100×, 3 within 300×, 5 within 1000×, and 2 within 10 000×). These margins might have been expected to be sufficient to yield a negative TQT result.

**Figure 2.**
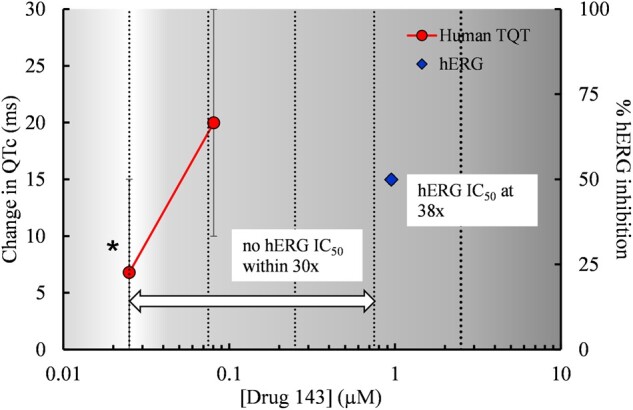
For Drug 143, concentration-response data from the clinical T thorough QT (QT) and nonclinical assays were plotted. The concentration-effect plots show data for Drug 143 used in the HESI/FDA analysis by Park *et al.* (2018). Stars indicate concentrations at which a positive effect occurred in the human TQT study. Although Drug 143 was positive in the human ether-a-go-go related gene (hERG) assay, this drug was classified as nonconcordant since the hERG IC_50_ value was beyond the 30× threshold margin established for concordance in the analysis by Park *et al.* (2018). The use of a larger safety margin (45×) as suggested by Gintant (2011) would have resulted in this drug being classified as concordant. The vertical clinical reference concentration (CRC) line indicates the lowest *C*_max, free_ showing QT prolongation. The additional vertical lines indicate multiples from the CRC. Data are plasma concentrations of unbound drug.

It is possible that the hERG assay/data itself may not have been conducted appropriately and some of these drugs could have been considered concordant with the positive TQT finding had the threshold been set at a greater multiple (eg, IC_50_ within 100×–300× CRC). It could be argued that 30-fold is not the most appropriate threshold to evaluate translation. An analysis by Gintant (2011) suggested that the predictivity of the hERG assays is better at 45-fold clinical exposures. Two of the 3 drugs causing clinical QT prolongation (ΔΔQTc) ≥20 ms had hERG IC_50_ values above the 30-fold threshold. An example is shown in Figure 2 for Drug 143, which had an hERG IC_50_ value at approximately 40-fold the positive clinical TQT study concentration and was therefore classified as discordant using the 30-fold threshold criterion. These examples highlight that a 30-fold margin between hERG IC_50_ and the clinical concentration does not always ensure lack of a clinical QT effect.

#### Electrophysiological mechanisms unrelated to hERG block

**Figure 3.**
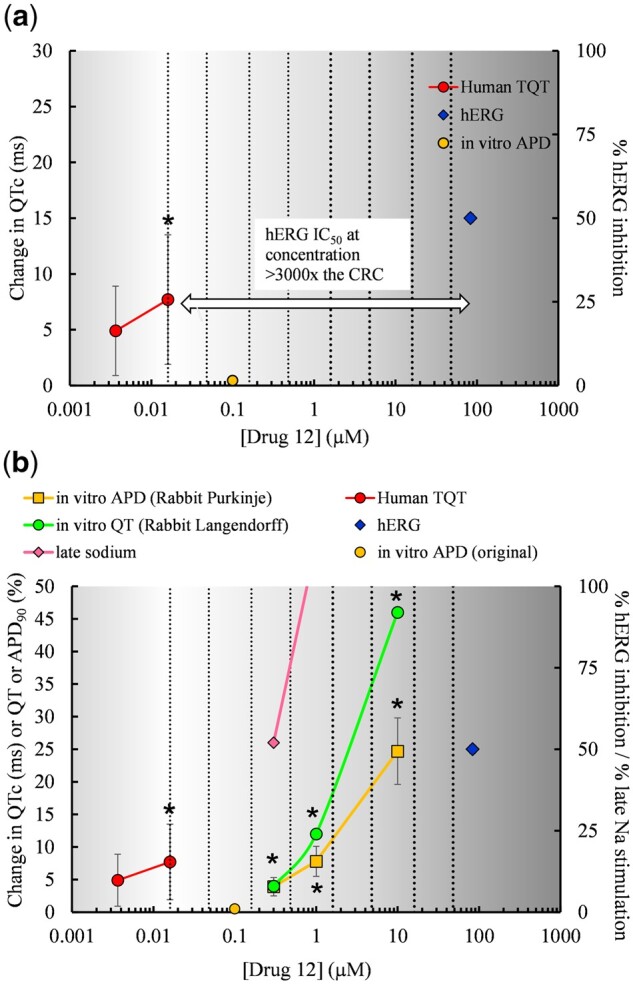
For Drug 12, concentration-response data from the clinical thorough QT (TQT) and nonclinical assays were plotted. The concentration-effect plots show (A) original clinical and nonclinical data for Drug 12 used in the Health and Environmental Sciences Institute/Food Drug Administration analysis by Park *et al.* (2018), and (B) data for Drug 12 with additional nonclinical data provided by the sponsor and from Lacerda *et al.* (2008). Stars indicate concentrations at which a positive effect occurred in each assay. TQT data are ΔΔ corrected QT (QTc). *In vitro* action potential duration (APD) data are from a rabbit Purkinje fiber study (APD_90_% change from control), and *in vitro* QT data are from a rabbit Langendorff study (QT% change from control). Patch clamp data indicating that Drug 12 increased the amplitude of the late sodium current (*I*_NaLate_) is included. At 10 µM the current is increased 396% but is cropped from the *y*-axis for display purposes. The supplementary nonclinical data from the sponsor and Lacerda *et al.* (2008) reveal a mechanism that produces QT prolongation but is not related to hERG block. These data render Drug 12 concordant with the human TQT study. The vertical clinical reference concentration (CRC) line indicates the lowest *C*_max, free_ showing QT prolongation. The additional vertical lines indicate multiples from the CRC. Data are plasma concentrations of unbound drug.

Although there is a strong association between inhibition of the hERG channel current and QT prolongation (De Bruin *et al.*, 2005; Gintant, 2011), drug effects at other ion channels can also affect the QT interval, so it is possible that discordance may have been due to non-hERG-mediated QT increases detected only in the clinical TQT study. Such an example is demonstrated in Figure 3A. Drug 12 caused a mean increase of 7.7 ms in QTc with an upper 95% confidence interval > 10 ms in the TQT study, despite there being no positive effects in the hERG assay within 3000-fold the clinical concentration or in the APD assay with 10-fold of the clinical concentration. No *in vivo* QTc data were available in the HESI/FDA database; however, subsequently generated nonclinical data (Lacerda *et al.*, 2008) has shown that Drug 12 (alfusozin, an α_1_ adrenoceptor blocker used to treat benign prostatic hyperplasia) may have the potential to prolong the QT interval. Drug 12 did not produce blockade of the cardiac repolarizing ion channels hERG, hKv4.3 (*I*_to_), or hKv7.1/KCNE1 (*I*_Ks_). However, alfusozin increased peak sodium (Nav1.5) current and increased the probability of late cardiac sodium channel current (*I*_NaLate_) openings (Lacerda *et al.*, 2008). Alfuzosin also reduced the slow time constant for sodium channel recovery from inactivation at a concentration of 300 nM (approximately 30-fold the *C*_max_). Data generated by Lacerda *et al.* (2008) also demonstrated APD_90_ prolongation with alfuzosin (300 nM) in a rabbit Purkinje fiber assay and QT prolongation in a rabbit Langendorff-isolated heart assay. A significant effect was observed in the Langendorff assay within 30× of the positive TQT concentration (Figure 3B). The Supplementary Material that was generated for alfuzosin makes the nonclinical and clinical data concordant and also revealed a non-hERG-mediated mechanism associated with QT prolongation.

### Indirect QT Effects

**Figure 4.**
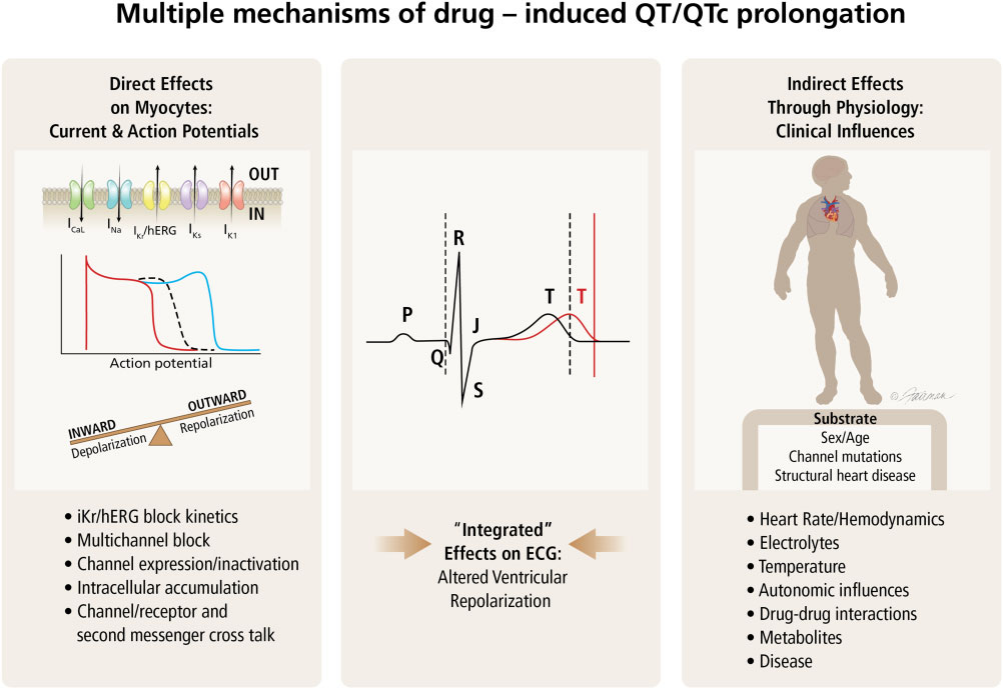
Multiple mechanisms may be involved in drug-induced QT/corrected QT (QTc) interval prolongation. The lack of concordance between human ether-a-go-go related gene (hERG) block (characterized by the IC_50_ value) and QT/QTc prolongation could be due to both direct and indirect factors. The left column lists various direct factors that may affect/modulate the extent of QT prolongation at the level of a myocyte beyond simple characterization of hERG block. These factors include the kinetics of hERG (*I*_Kr_) block, multichannel block (block of other channels that mitigate or exacerbate delayed repolarization, including transporters and electrogenic exchangers), drugs altering channel expression (“trafficking”), and intracellular accumulation and second messenger systems affecting ionic currents (eg, *I*_Ks_ current enhancement via cyclic AMP). The right column lists various indirect factors that may affect drug-induced QT prolongation including heart rate, electrolytes, and hemodynamic changes. Other factors that may affect the sensitivity of the myocyte to hERG block include hypokalemia-altered sympathetic/parasympathetic tone and diseases, such as heart failure. Note that the cardiac currents involved include the rapid component of the delayed cardiac potassium channel (Kv11.1 or *I*_Kr_,); the slow component of the delayed cardiac potassium channel (KvLQT1 + minK or *I*_Ks_); the L-type calcium channel (Cav1.2 or *I*_CaL_); the fast inward sodium channel (*I*_Na_); the inward rectifier potassium channel (Kir2.1 or *I*_K1_); the transient outward potassium channel (Kv4.3 or *I*_to_); and the late sodium channel (*I*_NaLate_). See the IUPHAR/BPS Concise Guide to PHARMACOLOGY citation for further details on these ion channels (Ion channels. Accessed on January 19, 2019. IUPHAR/BPS Guide to PHARMACOLOGY, http://www.guidetopharmacology.org/GRAC/FamilyDisplayForward?familyId=689).

**Figure 5.**
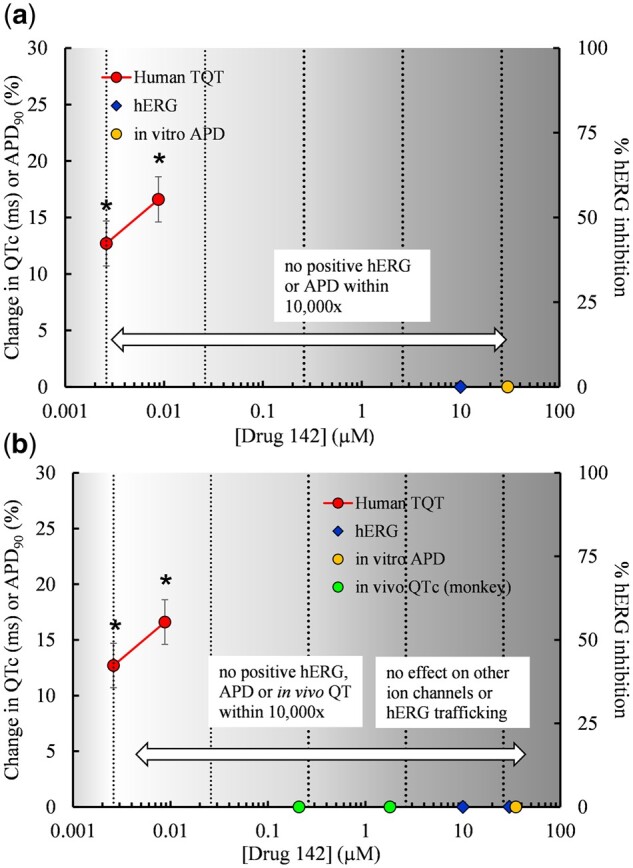
For Drug 142, concentration-response data from the clinical thorough QT (TQT) and nonclinical assays were plotted. The concentration-effect plots show (A) original clinical and nonclinical data for Drug 142 used in the HESI/FDA analysis by Park *et al.* (2018), and (B) data for Drug 142 with additional non-clinical data provided by the sponsor. Stars indicate concentrations at which a positive effect occurred in each assay. TQT data are ΔΔ corrected QT (QTc). The *in vitro* action potential duration (APD) is from a rabbit Purkinje fiber study (APD_90_ change from control). The *in vivo* QTc is from a monkey telemetry study. Supplementary Material failed to explain the increase in QT interval observed in the TQT study. The vertical clinical reference concentration (CRC) line indicates the lowest *C*_max, free_ showing QT prolongation. The additional vertical lines indicate multiples from the CRC.

The QT interval can be affected by many other drug-induced physiological changes (Figure 4). Several discordant TQT-positive drugs in the HESI/FDA dataset demonstrated no activity at hERG or other cardiac ion channels but may have produced physiological changes that indirectly caused changes in the QT interval. Drug 142, eg, caused QTc prolongation at both clinical doses tested in the TQT study, but did not show any activity in the hERG or APD assays at multiples of up to 10 000× the clinical concentrations (Figure 5A). The PK/PD relationship for Drug 142 showed a time delay between maximal plasma concentrations and observed QTc prolongation, which could not be explained by the formation of major metabolites. A second TQT study was performed at the supratherapeutic dose that confirmed the previously observed positive effect. Supplementary Material for this drug, provided by the sponsor, showed it had no effect on 13 other cardiac ion channels, no effect on hERG trafficking, and no effect on the QTc interval in a monkey telemetry study (Figure 5B). The mechanism for the increase in QT interval and resulting lack of concordance with Drug 142 has not been established; however, the drug caused hyperglycemia and bradycardia in clinical studies, and these may have had effects on the QTc interval.

**Figure 6.**
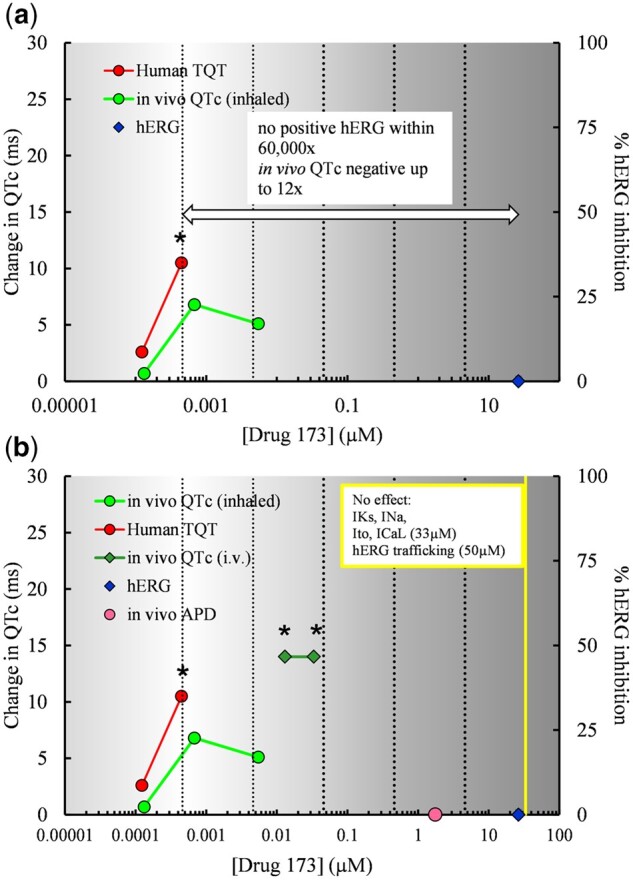
For Drug 173, concentration-response data from the clinical thorough QT (TQT) and nonclinical assays were plotted. The concentration-effect plots show (A) original clinical and nonclinical data for Drug 173 used in the HESI/FDA analysis by Park *et al.* (2018), and (B) data for Drug 173 with additional nonclinical data provided by the sponsor. Stars indicate concentrations at which a positive effect occurred in each assay. Both the TQT and *in vivo* corrected QT (QTc) data are ΔΔQTc. The *in vivo* QTc data are from dog telemetry studies where Drug 173 was given by both inhalational and intravenous routes of administration. *In vivo* action potential duration (APD) data are also shown from a guinea pig study. The supplementary nonclinical data provided by the sponsor for Drug 173 resulted in concordance with the clinical TQT data. The mechanism responsible for QT prolongation is considered indirect and may be related to a coincident increase in heart rate noted in both humans and dogs. The vertical clinical reference concentration (CRC) line indicates the lowest *C*_max, free_ showing QT prolongation. The additional vertical lines indicate multiples from the CRC. Data are plasma concentrations of unbound drug.

Drug interactions with targets involved in autonomic control may be a reason for the discordance of nonclinical and clinical repolarization assays. Drug 173 from the HESI/FDA database is 1 such example. Testing positive in the TQT study, Drug 173 showed no effects in the hERG assay up to 60 000× the CRC. It also produced no effects in the *in vivo* dog telemetry study up to 12× the CRC. Drug 173 was therefore classified as disconcordant (Figure 6A). Supplementary Material provided by the sponsor showed Drug 173 to have no effects in an anesthetized guinea pig assay (up to 4000-fold the CRC) and no effects at other cardiac ion channels including hKv4.3 (*I*_to_), hKv7.1/KCNE1 (*I*_Ks_), Nav1.5 (*I*_Na_), Cav1.2 (*I*_CaL_), and hERG trafficking (Figure 6B).

Drug 173 did, however, cause QTc prolongation in a follow-up dog QT telemetry study that involved intravenous administration of the drug instead of drug inhalation and produced higher concentrations (30×–100×) than the original study. These findings would result in Drug 173 being reclassified as concordant. The mechanism responsible for the QT prolongation is not fully understood, but the drug target is likely to affect autonomic control because, in both the TQT study and the follow-up dog telemetry study, QT prolongation occurred alongside tachycardia. This was marked in the dog telemetry study, and it has been suggested that the nonclinical QTc prolongation is secondary to the altered heart rate and/or changes in autonomic tone. In the TQT study, there was a linear PK/PD relationship with a QTc prolongation of 11 ms at the supratherapeutic dose (4× the clinical dose), but the increase in heart rate was not marked (≤ 8 bpm.)

## LIMITATIONS OF THE HESI/FDA DATABASE

It is important to highlight the discussion by Park *et al.* (2018) on the limitations of the HESI/FDA database drug analysis. This analysis compared clinical TQT study findings to results from the nonclinical hERG, APD, and *in vivo* QTc assays. One important limitation was the fact that the drug findings were either positive or negative in the APD and *in vivo* QTc assays, as interpreted by the study sponsors. Also, drug bath concentrations in the *in vitro* studies were often not measured to make it possible to attribute the changes observed to measured drug concentrations. Another limitation was that the range of free drug exposures in the nonclinical assays did not usually achieve the desired 1- to 100-fold multiple associated with the CRC. As well, not every drug that was included in the database was tested in each nonclinical assay, which creates smaller group sizes and limits evaluation. There was also bias in the dataset because sponsors typically submit drugs that lack or have limited effects on the QTc interval; there was a paucity of information regarding study quality, and a lack of additional nonclinical and clinical information to aid data interpretation. Last, the nonclinical studies submitted by the sponsors were markedly heterogeneous in nature. Some studies were conducted prior to implementation of the ICH guidelines and others were not robust, making the results difficult to generalize. This last limitation highlighted the need for greater standardization of experimental drug safety assays (Park *et al.*, 2018), and this point will form 1 of the pillars of the ongoing ICH Implementation Working Group discussion.

## THEORETICAL REASONS FOR THE LACK OF CONCORDANCE OBSERVED BETWEEN NONCLINICAL (HERG, APD, AND *IN VIVO* QT) ASSAYS AND CLINICAL QT/QTC PROLONGATION

In addition to those mechanisms for discordance highlighted in the case studies, there are numerous other factors that affect ventricular repolarization and QT interval and can cause discordant results between nonclinical and clinical data.

### Drug-Induced Multichannel Block

The alfuzosin case-study highlights the fact that QT prolongation in human studies likely results from the study drug interacting with ion channels other than hERG. It is also possible that hERG-blocking activity can be offset by effects at other ion channels (usually *I*_CaL_ or *I*_Na_) at similar concentration ranges. Mixed cardiac ion channel inhibition may occur for a molecule, but comprehensive *in vitro* testing of these properties has not been routinely conducted. Verapamil (hERG IC_50_ = 250 nM; Cav IC_50_ = 200 nM) and clozapine (hERG IC_50_ = 2300 nM; Cav IC_50_ = 3600 nM) are both potent hERG channel blockers but lack effects on repolarization in humans due to their simultaneous block of L-type calcium channels (Cavero and Crumb, 2001; Hoffmann and Warner, 2006; Kramer *et al.*, 2013). Ranolazine is an approved drug for the treatment of angina and is another example of a multiple-ion channel blocker (hERG IC_50_ = 6490 nM; Nav-late IC_50_ = 7884 nM) that is not arrhythmogenic, despite causing QT-prolongation due to hERG inhibition. If only hERG channel function is preclinically assessed, discordant repolarization data will likely occur. Assessment of a drug’s effects at multiple ion channels will more accurately predict the potential to prolong QT interval (Kramer *et al.* 2013) and its arrhythmogenic liability.

In contrast, vanoxerine is an example of an hERG-blocking drug that appeared to be safe based on its mixed ion channel activity, effects on APD in cardiac tissue and stem cells, and lack of arrhythmogenic signals from *in silico* simulation models (Obejero-Paz *et al.*, 2015; Piccini *et al.*, 2016). Unfortunately, although it was useful as an antiarrhythmic drug for the treatment of atrial fibrillation/atrial flutter, its use was associated with a significant risk of proarrhythmia (ventricular fibrillation/tachycardia requiring intervention, or TdP) in patients with structural heart disease. Mixed ion channel blocking drugs must be carefully evaluated for potential arrhythmogenic liability in the context of their intended patient population, which may have underlying CV comorbidities (Lacerda *et al.*, 2010).

### The Kinetics of *I*_Kr_ (hERG) Block

The primary molecular mechanism for drug-induced QT interval prolongation is direct inhibition of the voltage-gated potassium channel that generates the rapid component of the delayed rectifier potassium current (*I*_Kr_) (Figure 4). hERG (KCNH2, K_V_11.1) is the human ether-à-go-go-related gene (hERG) that codes for the α-subunit of this ion channel. The *in vitro* hERG assay is typically performed with stable mammalian cell lines (either human embryonic kidney [HEK] cells or Chinese hamster oocytes [CHO]) that only express the α-subunit (Gamper *et al.*, 2005; Jayapal *et al.*, 2007; Thomas and Smart, 2005). Several complicating *in vivo* factors must be considered when evaluating data generated by an *in vitro* hERG assay, including ancillary subunits other than the α-subunit (Anantharam and Abbott, 2005); plasma membrane-associated protein KCR1 and reduced sensitivity to hERG block (Kupershmidt *et al.*, 2003); and regional variation in molecular expression in the heart (Anantharam and Abbott, 2005; Antzelevitch, 2007).

*In vitro* IC_50_ values are used to characterize hERG channel blockade and are the preferred way to estimate drug effects on repolarization. It is worth noting that drug-induced QTc prolongation *in vivo* can occur at free plasma concentrations in the range of hERG IC_10_ (Takasuna *et al.*, 2009), and IC_10_ or IC_50_ values represent an oversimplification of complex time-, voltage- and state-dependent processes *in vivo*.

There are many potential nonion channel-related mechanisms that could cause QT interval prolongation (Figure 4). These include membrane proteins (eg, Na/Ca exchanger, Na/K-ATPase, Na/H exchange, and ATPase) resulting in changes to cellular electrolyte homeostasis, intracellular calcium levels, cardiac resting membrane potential, and cell volume and intracellular sodium and pH (Iwamoto, 2007; Pierre and Xie, 2006; Karmazyn, 2013).

#### Drug-induced protein trafficking inhibition of *I*_Kr_ channel expression

Drug effects on hERG protein trafficking, from the site of synthesis at the endoplasmic reticulum (ER) to the active location of the protein at the cell surface, are well-described. Ion channels may undergo relatively rapid turnover, with estimated half-lives on the order of hours in cultured cells (Takimoto *et al.*, 1993). Wible *et al.* (2005) showed that up to 40% of the hERG blockers evaluated also inhibited hERG protein trafficking in cardiac cells; but most importantly, approximately 6% of non-hERG blockers specifically altered hERG trafficking. ER-located chaperone proteins (such as heat shock proteins [Hsp] 70 and 90) are critical for correct hERG channel expression. Drugs that inhibit chaperone function produce a novel form of acquired long-QT syndrome, caused by the reduced surface expression of channels from a defect in either the forward (toward cell membrane) or backward (away from cell membrane) trafficking mechanisms required for hERG channel assembly (de Git *et al.*, 2013; Wible *et al.*, 2005).

Arsenic trioxide was the first example of a therapeutic drug that caused QT prolongation, TdP, and sudden death by reducing the formation of the hERG-chaperone complexes Hsp 70 and Hsp 90 (Ficker *et al.*, 2004; Nogawa and Kawai, 2014). Another drug that inhibits hERG trafficking is geldanamycin, which inhibits Hsp70 and increases the degradation of the protein, and pentamidine that arrest hERG protein folding (Nogawa and Kawai, 2014), prevents maturation, and disrupts transport from the ER (Kuryshev *et al.*, 2005; Nogawa and Kawai, 2014). The cholesterol-lowering drug probucol is another example of a medication that causes QT interval prolongation and TdP in patients (Tamura *et al.*, 1994) by attenuating cellular caveolin-1 turnover and accelerating mature hERG channel degradation (Guo *et al.*, 2011).

The effects of drugs on the trafficking of other cardiac ion channel proteins are largely unknown, but cisapride can cause QT prolongation by rescuing an abnormal misprocessed sodium channel protein called SCN5A and inhibiting hERG block (Liu *et al.*, 2005).

#### The effect of intracellular accumulations of drugs on *I*_Kr_

Some drugs cause QT prolongation at lower concentrations than predicted by the hERG assay; perhaps via intracellular accumulation. Most comparisons of *in vitro* and *in vivo* data are based on free (or total) drug plasma concentrations, and little attention has been paid to the kinetics of drug/metabolite uptake into the myocardium, despite the well-known adverse effects of myocardial accumulations of QT-prolonging drugs such as the antihistamine terfenadine and antipsychotics such as haloperidol and olanzapine (Cavero and Crumb, 2001; Titier *et al.*, 2004).

Minematsu *et al.* (2001) investigated the time-dependent relationship between the myocardial concentration of tacrolimus and the development of QT prolongation in guinea pigs. The distribution of tacrolimus in the myocardium was delayed, eventually reaching a 50-fold higher concentration in tissue than in blood. Myocardial concentration remained high after the end of the infusion period. The magnitude of QT prolongation paralleled the time-dependent concentration of tacrolimus in ventricular tissue, but not in plasma.

Chloroquine and hydroxychloroquine are traditionally used for the treatment of malaria and have recently gained interest and attention in the setting of the 2019 novel coronavirus (Delaunois *et al.*, 2021). Both drugs are associated with hERG inhibition, QT prolongation, and a risk of arrhythmias/TdP (Anon., 2016; WHO, 2016). Furthermore, both drugs have been shown to have higher concentrations in the heart than the plasma, which may contribute to their adverse CV profiles, particularly in patients with CV-associated comorbidities (Adelusi and Salako, 1982; McChesney *et al.*, 1967). There is no clear evidence about whether either of these drugs is actively transported into the heart, and limited information exists on the functional role of efflux transporters in the heart. Some evidence shows that P-glycoprotein acts as a drug efflux pump and can decrease the cellular concentration of a drug and protect the heart (Colombo *et al.*, 1996; Weiss and Kang, 2002).

#### The effect of ion channels, receptors, enzymes, transporters, second messenger cross-talk, and autonomic effects on *I*_Kr_

Cardiac ion channels in the sarcolemmal membrane function within macromolecular complexes that are influenced by protein-protein interactions. These interactions may involve channel accessory subunits, kinases, phosphatases, scaffolding proteins, cytoskeleton proteins, and receptors (Kagan and McDonald, 2005). Modulating proteins in the sarcolemmal membrane could modify *I*_Kr_ activity.

Drug channel/receptor cross-talk cannot be detected *in vitro* using the hERG assay in transfected HEK/CHO cells because they have a limited number of receptors and second messenger systems that are also expressed in native mammalian cardiac myocytes. This problem highlights the need to perform secondary pharmacology screening assays against multiple receptors, ion channels, transporters, and enzymes to fully understand possible interactions with *I*_Kr_ (Bowes *et al.*, 2012; Lynch *et al.*, 2017; Valentin *et al.*, 2018). In general, *in vivo* QT assays should be used to detect these interactions, with particular attention to differences in receptor expression between species of animals and between animals and humans.

There is growing evidence for a relationship between hERG activity and the sympathetic nervous system, making adrenergic receptor interactions with hERG of special interest. In anesthetized rabbits, TdP can only be reproduced when there is concomitant treatment with the α_1_-adrenoceptor agonist methoxamine (Carlsson *et al.*, 1993). Functional coupling of α-adrenoceptors to hERG has been described in several studies (Bian *et al.*, 2001; Thomas *et al.*, 2004), suggesting that the mechanism of interaction occurs at the level of intracellular signaling pathways. Protein kinase A (PKA), protein kinase C, and phosphatidyl-4,5-biphosphate appear to play a role (Bian *et al.*, 2001; Thomas *et al.*, 2004). Other studies have revealed that high levels of catecholamines are probably associated with stressor responses acting through β-adrenoceptors, and they modulate *I*_Kr_ via PKA (Karle *et al.*, 2002). This results in complex interactions between PKA, intracellular messengers, and hERG (Cui *et al.*, 2000; Hoffmann and Warner, 2006).

β-adrenoceptor agonists have also been shown to induce QTc prolongation in humans, despite having no effect on hERG, Purkinje fiber APD, or *in vivo* dog QTc assays (Newbold *et al.*, 2007). Several studies have described the effects of adrenergic stimulation on QT/repolarization in dogs via direct agonism with isoproterenol or phenylephrine, or indirectly via nitroprusside-induced hypotension with reflex activation of the peripheral sympathetic tone—alone and in the presence of hERG blockade (Fossa *et al*, 2005, 2007).

The data show the impact of autonomic alterations and their influence on the temporal heterogeneity and hysteresis of cardiac restitution, which can be an additional arrhythmogenic substrate. Additional clinical evidence shows a clear interaction between autonomic function, cardiac electrophysiology, and arrhythmogenicity.

The congenital long-QT syndromes (LQT1, LQT2, and LQT3) are associated not only with QT prolongation but also ventricular arrhythmias that are often triggered by changes in autonomic tone via a stress/startle response to increase sympathetic tone (LQT1 and LQT2) or sleep/bradycardia associated with relatively higher parasympathetic or vagal tone (LQT3; Schwartz *et al.*, 2001). In fact, management of these genetic forms of QT-prolongation includes treatment with β-blockers and, in some cases, surgical cardiac sympathetic denervation (Schwartz *et al.*, 2012). Taken together, available data suggest that stimulation of cardiac G protein-coupled receptors can modulate *I*_Kr_-dependent repolarization.

Although *I*_Kr_ is the primary cardiac ion channel that modulates cardiac repolarization there are additional K^+^ channels that are intimately involved in repolarization and should be considered since the hERG assay alone does not address all drug effects that may be responsible for QT prolongation (Lu *et al.*, 2008; Towart *et al.*, 2009). Voltage-gated channels such as the transient outward (*I*_to_), the slow delayed rectifier (*I*_Ks_), and the inward rectifier (*I*_K1_) K^+^ currents and their many subtypes have marked influence on repolarization. The rapid voltage sensitive activation and inactivation of *I*_to_ contribute substantially to the very early phase of repolarization prior to the action potential (AP) plateau, whereas *I*_Ks_ is involved in late repolarization. The inward rectifier (*I*_K1_) K^+^ channel is strongly active at potentials close to the resting membrane potential and therefore is vital to both rapid initial depolarization and terminal repolarization and maintenance of the cardiac resting membrane potential. Zhao *et al.* (2012) showed that a reduction in the cardiac AP plateau voltage by *I*_to_, in the presence of reduced repolarization reserve (ie, reduced *I*_Ks_), may precipitate the development of voltage oscillations manifest as early afterdepolarizations. Lengyel *et al.* (2007) also showed that the addition of *I*_Ks_ blockade to pre-existing *I*_Kr_ blockade produces additive QT prolongation, and TdP arrhythmias. Jalife (2009) implicates *I*_K1_ as a stabilizer of reentry during VF because of its ability to shorten the APD and affect cardiac conduction velocity by reducing the cardiac wavelength and enhancing membrane hyperpolarization. These effects contribute to a greater availability of Na channels during the excitable gap of reentry which increases excitability perpetuating VF. Thus, prolongation of ventricular repolarization that is often associated with *I*_Kr_ emphasizes consideration of other cardiac K^+^ channels in the absence of drug-induced *I*_Kr_ blockade.

### Indirect Effects on Myocytes: Substrate and Clinical Influences

Although assessing the direct effects of an NCE on cardiac electrophysiology and function is critical to drug safety, it is important to consider the indirect effects that an NCE may also have on the heart and CV system. This section considers other factors that may prolong the QT interval.

#### Influence of gender and Age on the QT interval

Women, compared with men, are at a greater risk for developing drug-induced QTc interval prolongation and subsequent arrhythmias (Abi-Gerges *et al.*, 2004; Yang *et al.*, 2011). The gender difference in susceptibility to QTc prolongation may result from the physiological effects of sex hormones (Shuba *et al.*, 2001) or to altered expression levels of cardiac ion channels (Ridley *et al.*, 2008; Surawicz, 2001). The QTc interval is similar between genders in children younger than 15 years of age prior to the onset of puberty; however, after puberty the QTc interval decreases in males and increases in females (Ramirez *et al.*, 2011). This androgen-driven difference (Lazarus, 2001) is constant until males reach the age of approximately 55, when male testosterone levels decline. Elderly men and women tend to have longer QTc intervals than nonelderly adult men and women (Khan *et al.*, 1998). Despite these clinical observations, nonclinical QT studies are usually performed in sexually mature male animals. Because of this tendency, gender-specific effects are not likely to be detected in standard nonclinical investigations, so age as well as gender should be considered in CV safety studies.

#### Influence of ion channel mutations on the QT interval

The potencies of drugs blocking hERG are commonly assessed using cell lines that express the human wild-type variant. Many clinically diagnosed cases of long QT syndrome can be genotyped. Twenty to forty percent of them have mutations in the *KCNH2* gene that encodes the hERG alpha subunit of the channel conducting the *I*_Kr_ current (Larsen *et al.*, 2001; Wang *et al.*, 1996). Männikkö *et al.* (2010) tested whether potencies for hERG channel inhibition would be different for hERG single nucleotide polymorphisms (SNPs). These *in vitro* studies showed that the expressed hERG channel is not significantly affected by any of the 9 common SNPs produced by 50 marketed drugs. Since the amino acid change for each of the investigated SNPs is not located on or near the putative drug binding site, these *in vitro* findings are not surprising (Mitcheson and Perry, 2003); but data suggests that amino acid changes remote from drug binding sites *can* affect hERG. For example, an SNP (Q9E) in MinK-related peptide 1, the putative β subunit of *I*_Kr_, has been shown to increase the potency of hERG block by clarithromycin (Abbott *et al.*, 1999).

#### Presence of structural heart disease on the QT interval

Studies that are conducted to evaluate the safety profile of an NCE use healthy animals for the nonclinical program and healthy volunteers for early clinical studies. In contrast, marketed drugs are likely to be given to patients with various stages of heart disease. Investigators and clinicians should remember that acquired QT prolongation is a common electrocardiogram (ECG) finding in people with heart disease.

In animal models of cardiac hypertrophy and failure, prolongation of the APD is a consistent finding (Aronson, 1981; Tomaselli *et al.*, 1994). Disturbed ventricular repolarization was found in the canine TdP arrhythmia model with chronic, complete atrioventricular block (Volders *et al.*, 1999). In fact, both the ventricular endocardial monophasic AP and QT interval were prolonged in these dogs, and a significant downregulation of delayed rectifier K currents (both *I*_Kr_ and *I*_Ks_) was observed. This downregulation existed at both the level of the ionic current and the expression of mRNA and protein, indicating that acquired QT prolongation and TdP in this model are related to intrinsic repolarization defects, also called electrical remodeling of the ventricles.

In the failing human heart, there is a downregulation of potassium channels that contributes significantly to the enhanced liability of the repolarization process (Nabauer and Kaab, 1998). Vanoxerine (highlighted above) is an example of a drug in which potential adverse cardiac effects can be different in healthy and diseased hearts. Vanoxerine is an orally active, mixed ion channel blocker with *I*_Kr_, *I*_Na_, and *I*_CaL_ activity that is effective for the termination of recent-onset atrial flutter and atrial fibrillation. It does not produce adverse cardiac events in normal volunteers; but in patients with structural heart disease, its use was associated with a significant risk of ventricular proarrhythmia (Christophe and Crumb, 2019; Piccini *et al.*, 2016).

#### Effect of heart rate and autonomic tone on the QT interval

Heart rate effects can either be direct on pacemaker cells or indirect, either through vasodilation and the resulting reflex tachycardia or other mechanisms that affect autonomic tone. Because the QT interval adapts to changes in heart rate, it is difficult to compare QT intervals recorded at different heart rates. In fact, the QT interval and heart rate have an inverse, nonlinear relationship, which varies among species and between animals within a species, so drug effects on the QT interval should be interpreted with concomitant changes in heart rate in mind.

The most common approach to correct the QT interval for heart rate (QTc) involves the use of a correction formula such as those developed by Bazett or Fridericia in 1920. These formulas attempt to normalize observed QT intervals for the time interval between 2 successive R-waves of the QRS complex an interval of 1000 ms (60 beats/min) into comparable referenced QTc values. Many mathematical formulas that involve both linear and nonlinear regression analyses have been created over the years to correct the QT/RR interval more accurately; but none of them can completely compensate for the variations produced by drug effects on the actual QT interval if physiological heart rate changes occur outside of the baseline QT/RR interval range used to define the correction formula. Furthermore, the correction formulas may not be effective for assessing the risk of QT interval prolongation when drug effects on heart rate are large.

An alternative approach is to correct the QT interval using formulas applied to values derived from individual animals, or to analyze the QT/RR relationship. Fossa *et al.* (2005) suggested a dynamic assessment of the beat-to-beat QT/RR interval relationship. This technique studies beat-to-beat heart rate variability, hysteresis of the QT/RR relationship, and QT interval heterogeneity. It also provides a way to differentiate QT interval prolongation effects produced by hERG blockade from changes in the QT interval produced by autonomic-mediated reflexes and avoids the errors associated with the use of standard correction factors, such as those of Bazett and Fridericia.

Additional studies have shown that drug-induced effects on autonomic tone can affect the QT interval. This is exemplified by the findings on the phosphodiesterase type 5 inhibitors sildenafil and vardenafil that block the enzyme responsible for degrading cyclic GMP in vascular smooth muscle cells. These drugs are relatively weak hERG blockers compared with their respective clinical free therapeutic plasma exposure levels, with safety margins of ≥ 1000. However, these drugs show slight QT interval prolongation in humans (up to approximatley 8–10 ms; Alpaslan *et al.*, 2003; Fossa *et al.*, 2011) at clinical doses that produce mild vasodilation and reflex tachycardia. Fossa *et al.* (2011) demonstrated that enhanced QT analysis methods were required to evaluate drug-induced changes in repolarization when changes in heart rate or autonomic tone also occur. A beat-to-beat analysis (QTbtb) showed that the upper bounds of the QTbtb was < 8 ms for vardenafil, while the use of standard correction methods such as QTc_F_ resulted in changes of > 10 ms. Other methodologies to assess QT interval prolongation alongside changes in heart rate and autonomic tone have also been described (Garnett *et al.*, 2012), including analysis of uncorrected QT interval bins over specified RR interval bins (Holter bin analysis) for a direct comparison of drug versus vehicle/placebo at comparable heart rates.

Although QT interval duration varies directly with changes in the RR interval, QT interval rate-adaptation is not precisely regulated on a beat-to-beat basis but is strongly influenced by the preceding heart rate history. This well-known and problematic phenomenon is referred to as QT hysteresis (Pueyo *et al.*, 2003). To address this problem, Holzgrefe *et al.* (2007) suggested a probabilistic QT rate-correction method and definition for termination of the T-wave (*T*_end_) that allowed for the derivation of a stable and sensitive QTc measure in dogs and cynomolgus monkeys. Their work also indicates that probabilistic raw QT interval determinations are equally applicable to human ECG analysis.

Recently, Malik *et al.* (2018) used a statistical modeling approach to estimate drug-induced QTc changes at mean maximum plasma concentrations of investigated drugs. They found that correction for QT/RR hysteresis should be incorporated into studies since corrections that omit consideration for hysteresis may lead to erroneously biased conclusions and false positives (Malik *et al.*, 2018).

#### Effects of electrolytes on the QT interval

Electrolyte disturbance because of drugs or disease (ie, hypokalemia) can produce a number of effects on cardiac electrical activity due to altered transmembrane potentials that ultimately lead to changes in ionic permeability and conductivity. The cardiac cell membrane has distinct ion channels and the resulting resting membrane potential is due to a unique balance between the equilibrium potentials for the various ions (Gintant *et al.*, 2016). Thus, changes in serum electrolytes can alter cardiac ion currents as well as channel kinetics which may be pro- or antiarrhythmic. Potassium is the most abundant intracellular electrolyte and is primarily responsible for establishing cell membrane potential (*V*_m_), so maintaining potassium homeostasis is critical to preventing the development of severe adverse cardiac events (Trenor *et al.*, 2018).

Clinically, hypokalemia is most commonly encountered when people take β_2_-adrenoceptor agonists (Cazzola *et al.*, 2011) and diuretics that both alter the renal regulation of potassium (Duke, 1978) and enhance drug-induced prolongation of the QT interval (El-Sherif and Turitto, 2011; Weiss *et al.*, 2017). ECG changes during hypokalemia are manifest as inverted or flat T-waves, ST segment depression, and prominent U-waves (Gennari, 1998). Studies have shown that hypokalemia can alter *I*_Kr,_ increase channel inactivation, slow the reactivation kinetics of the transient outward potassium current (*I*_to_), and cause *I*_Kr_ channels to become nonconducting (Weiss *et al.*, 2017). Melgari *et al.* (2014) showed that hypokalemia may impair the channel's protective role against premature excitation, so hypokalemia can augment proarrhythmic risk in the presence of drugs that block *I*_Kr_.

Hyperkalemia due to reduced kidney function; hyperaldosteronism; or the use of drugs such as spironolactone (a diuretic), certain antihypertensive drugs (angiotensin-converting enzyme inhibitors), or nonsteroidal antiinflammatory drugs cause a depolarization of cardiac cell membrane potential. This shortens the APD, changes cardiac conduction velocity (Weiss *et al.*, 2017), and produces peaked T-waves, a broad P-wave, and a wide QRS interval in the ECG due to slowed conduction (Weiss *et al.*, 2017). Hyperkalemia also causes sodium and calcium channels to open, increases potassium channel conductance, and slows the inactivation time-constant of the hERG channel (Melgari *et al.*, 2014), which may encourage early repolarization (the development of J-waves), a shortened QT interval, and increased tissue sensitivity to arrhythmias (Melgari *et al.*, 2014).

Alterations in calcium can have severe effects on cardiac function and cellular repolarization processes. Hypocalcemia due to kidney failure or hypoparathyroidism causes both negative inotropy and chronotropy, which results in cardiac instability and an increase in TdP risk. If hypocalcemia is marked, the effect can mimic acute myocardial ischemia (El-Sherif and Turitto, 2011). Hypercalcemia increases heart rate and causes positive inotropy but shortens the QT interval of the ECG.

Unlike potassium and calcium, limited electrophysiological effects are observed from changes in serum sodium or other electrolyte abnormalities (El-Sherif and Turitto, 2011). Hypomagnesemia is associated with an imbalance in sodium, potassium, or calcium electrolytes and can lead to coronary artery vasospasm, arrhythmias, ischemic damage, and cardiac failure (Chakraborti *et al.*, 2002).

#### Influence of changes in body temperature on the QT interval

It is well known that core body temperature changes can affect the QT interval. Many drugs including opioids, antihistamines, anticholinergics, anesthetic drugs, antidiabetic medications, and tricyclic antidepressants can modulate body temperature (Cuddy, 2004). The hypothalamus maintains the set point for body temperature through reflexes that cause vasodilation and sweating when the body is too warm, or vasoconstriction and shivering when the body is too cold (Morrison and Nakamura, 2011). Clinically, QTc interval prolongation occurs repeatedly with hypothermia treatment. In 1 patient, the QTc interval was 549 ms (at 37°C) prior to the onset of hypothermia and prolonged to 686 ms at a body temperature of 32°C, ie, a 25% increase (Huang *et al.*, 2006).

Nonclinically, hypothermia produces a lengthening of the cardiac AP but also decreases the diastolic membrane potential in isolated guinea pig ventricular muscle (Lathrop *et al.*, 1998), and prolongations of the QT interval have been associated with decreases in body temperature in anesthetized beagle dogs (van der Linde *et al.*, 2008). Discordance between *in vitro* and *in vivo* repolarization assays can occur if hERG-inactive drugs cause changes in body temperature, and some drugs may be incorrectly classified as having direct effects on repolarization (QT) because of this. In addition, direct QT effects may be missed when testing drugs that change body temperature. Correction methods have been devised (van der Linde *et al.*, 2008) and successfully applied (El Amrani *et al.*, 2019) to correct QT simultaneously for changes in heart rate and body temperature.

Under *in vitro* conditions, temperature can influence the hERG inhibitory potency of drugs. Increases and decreases in temperature change the voltage dependence of channel kinetics (Kirsch *et al.*, 2004; Vandenberg *et al.*, 2006). Since temperature can change channel kinetics, the state dependence of drug binding changes as well, and this must be considered when determining drug block (ie, IC_50_ values). An increase in temperature from 22°C to 35°C caused some drugs to decrease blocking potency, some drugs to increase blocking potency, and had no effect on others (Kirsch *et al.*, 2004; Yao *et al.*, 2005). Currently, the hERG assay is conducted at temperatures at or near body temperature which is now best practice for conduct of this assay.

#### Drug-drug interactions and the QT interval

PD drug-drug interactions at the level of the hERG channel protein are not well understood. The high activity binding site of hERG blockers is located in the central cavity of the channel (Sanguinetti and Mitcheson, 2005). The large pore, together with the 4-fold symmetry and abundance of aromatic and polar regions that form the binding site, allows for the development of many different binding modes and therefore many different drug interactions. There is no conclusive experimental evidence for other potential binding sites to date (Sanguinetti, 2005) except for peptide toxins, which clearly bind to an external site (Jimenez-Vargas *et al.*, 2012). Allosteric interactions (such as binding a molecule outside the high-affinity binding site, which influence gating properties) may also influence hERG blocking properties. Wang *et al.* (2003) showed that saxitoxin, eg, binds to the hERG channel at multiple sites that can alter the energetics of channel gating, suggesting that an alternate channel gating mechanism exists when allosteric coupling takes place.

PK drug-drug interactions, especially at the cytochrome P450 level, can lead to clinically relevant prolongation of the QT interval. The 6 major enzymes of the cytochrome P450 system (CYP1A2, 2C9, 2C19, 2D6, 2E1, and 3A4) metabolize > 90% of existing drugs (Rendic, 2002). The most dramatic example of a PK drug-drug interaction is the antihistamine terfenadine, formerly used for the treatment of allergic conditions but removed from the U.S. market by the manufacturer in 1997. It is a prodrug, generally completely metabolized in the liver by the CYP3A4 enzyme to the hERG-inactive/pharmacologically active form, fexofenadine. Due to its near complete metabolism by the liver immediately after absorption from the gastrointestinal tract, terfenadine is normally present at very low systemic plasma concentrations; however, simultaneous treatment with other CYP3A4 substrates, such as ketoconazole or erythromycin, can inhibit terfenadine metabolism and increase systemic plasma concentrations to levels that inhibit hERG and result in QTc prolongation and TdP (Thompson and Oster, 1996).

QTc prolongation in the setting of antipsychotic drug use is well documented and another good example of the PK drug-drug interactions on drug safety. Many antipsychotics, especially first-generation drugs, warrant cardiac monitoring, and several antipsychotics have been withdrawn from the market due to their association with QTc prolongation and fatal TdP events (Vieweg, 2003). PK interactions by antipsychotics primarily involve the CYP2D6 and CYP3A4 enzyme pathways (Brown *et al.*, 2004). Antipsychotics involving the CYP2D6 isozyme adversely interact with tricyclic antidepressants, selective serotonergic reuptake inhibitors, and β-blockers. Among these drug interactions, tricyclic antidepressants were most likely to cause clinically significant QT changes because of their narrow therapeutic index. Drug interactions related to the CYP3A4 pathway mainly involve high-potency antipsychotics coadministered with inhibitors such as clarithromycin.

In a comprehensive study, the QT interval was examined for effects by newer antipsychotic drugs including ziprasidone, risperidone, quetiapine, and olanzapine and compared with thioridazine and haloperidol (Food and Drug Administration Advisory Committee, 2000). Although these drugs prolong the QTc interval, only quetiapine and haloperidol were associated with drug-induced QTc interval prolongation exacerbated by the presence of a metabolic inhibitor.

#### Major drug metabolites may affect the QT interval

According to the Metabolites in Safety Testing (MIST) guidelines, the effects of major drug metabolites that may or may not be pharmacodynamically active at the therapeutic site cannot be evaluated using *in vitro* assays (hERG or APD) unless the molecule is known, can be synthesized, and is subject to independent evaluation. However, in the *in vivo* QT assay, the effects of major metabolites may be detected, provided that relevant plasma concentrations of the major metabolite are reached within 24 h, which is the duration of standard pharmacology safety studies. Alternatively, QT effects can be evaluated in repeat-dose telemetry or general toxicology studies; however, there is always the possibility that the selected nonrodent species exhibits a qualitatively or quantitatively different metabolic profile than humans. It is also possible that a unique human metabolite with hERG inhibitory activity may not be formed in the selected preclinical species, and vice-versa.

Many aspects of drug metabolism should be considered when evaluating an NCE. For instance, while every tissue has the potential capability to metabolize drugs, the hepatocyte is the primary site for drug metabolism. The heart is generally considered to be metabolically inert. However, available information indicates that drug-metabolizing enzymes are expressed in the heart at the gene and protein level, and an increase in the cytochrome P450 level was noted following the induction of cytochrome P450 enzymes by Aroclor 1254 treatment (Thum and Borlak, 2000). The elevated cytochrome P450 levels in cardiomyocyte cultures were, however, markedly lower than those in hepatocyte cultures and Aroclor 1254 treatment resulted in lower induction rates in cardiomyocytes than in hepatocytes.

#### Glycemia and QT interval

It is possible that a prolonged QTc interval in type 2 diabetes mellitus (T2DM) patients is merely a surrogate biomarker representing multiple complex underlying pathophysiological processes including subclinical atherosclerosis, diabetic cardiomyopathy, and diabetic autonomic neuropathy (Cox *et al.*, 2014; Takahashi *et al.*, 2004). Vaghela *et al.* (2016) showed that 64% of T2DM patients showed prolonged QTc intervals. In another T2DM study, the QTc interval was an independent predictor of all-cause and CV disease mortality (Cox *et al.*, 2014); and changes in glycemic levels, either up or down, prolonged the cardiac AP and prolonged the QT interval (Kacheva *et al.*, 2017; Marfella *et al.*, 2000; Nordin, 2014).

Zhang *et al.* (2003) provide direct evidence that both hypoglycemia and hyperglycemia can inhibit the hERG channel *in vitro.* The inhibition of hERG results from the underproduction of ATP in hypoglycemia and the overproduction of reactive oxygen species in hyperglycemia. Ashrafi *et al.* (2017) found that, compared with controls, human patients with T2DM had downregulated left ventricular hERG gene expression that resulted in left ventricular hypertrophy, possible myocardial fibrosis, and QT interval prolongation. Modeling of the altered current densities in diabetic patients revealed a prolonged AP (Hancox, 2017).

Ventricular repolarization in overweight and obese subjects is of particular interest due to the high prevalence of metabolic disorder. A systematic review and meta-analysis of the effects of overweight/obesity on QTc by Omran *et al.* (2016) provided clear evidence that subjects who are overweight and obese have significantly prolonged QTc intervals. Weight loss in these subjects produced a significant reduction in the QT interval.

## EVOLUTION OF REGULATORY REQUIREMENTS IN CARDIAC SAFETY TESTING

Both ICH S7B (Anon., 2005a) and ICH E14 (Anon., 2005b), finalized in May 2005, described nonclinical and clinical risk assessment strategies, respectively, for QT interval prolongation and proarrhythmia, that can influence the design of clinical investigations. Although E14 originally suggested a QT interval evaluation independent of S7B results, both documents highlight the need for data integration for risk assessment. ICH S7B has remained unchanged since its adoption, but ICH E14 has undergone 3 reviews to clarify the incorporation of novel technologies: ECG monitoring in late-stage clinical trials; heart rate correction; QT assessment for drug combinations, large targeted proteins; monoclonal antibodies and, more recently, using concentration response modeling of QTc data in early clinical trials instead of the dedicated TQT study, although under tightened conditions.

Over the past several years, emergent data from the Comprehensive *in vitro* Proarrhythmia Assay (CiPA; https://cipaproject.org/about-cipa/, Strauss *et al.*, 2019) and the Japanese iPS Cardiac Safety Assessment (JiCSA) have helped the ICH update the S7B and E14 guidance documents (Asakura *et al.*, 2015; Blinova *et al.*, 2018).

In its current form, CiPA uses mechanistic electrophysiologic data to evaluate proarrhythmic risk (Fermini *et al.*, 2016; Sager *et al.*, 2014). This includes (1) an assessment of drug effects on multiple human cardiac ion currents, (2) *in silico* reconstruction of human ventricular electrophysiological activity (Li *et al.*, 2017; O’Hara *et al.*, 2011), (3) the use of human-induced pluripotent stem cell cardiac myocytes (hiPSC-CMs) to confirm the findings of the *in vitro* and *in silico* assays (Fermini *et al.*, 2016; Peng *et al.*, 2010; Pugsley *et al.*, 2015), and (4) the clinical evaluation of drug effects in human Phase I studies for potential unanticipated electrophysiologic ECG signals (Vicente *et al.*, 2019).

The JiCSA initiative (Kanda *et al.*, 2018) used multi-electrode array platforms (hiPSC- and iCell-CM) to assess QT interval prolongation and the proarrhythmic potential of drug (Yamamoto *et al.*, 2016). In collaboration with the *Japanese Safety Pharmacology Society*, JiCSA completed a large-scale validation study using 60 drugs with a variable incidence of TdP risk (Ando *et al.*, 2017; Kanda *et al.*, 2018). JiCSA also participated in the CiPA international multisite study and evaluated the 28 blinded CiPA drugs tested by the HESI/FDA consortium (Blinova *et al.*, 2018; Yamazaki *et al.*, 2018). The JiCSA results were in general agreement with the HESI/FDA data (Kanda *et al.*, 2018), and the studies provided important insights into the value of hiPSC-CMs and the methods required to justify their use in potential regulatory submissions.

The CiPA and JiCSA data also led to the view that regulatory guidance was needed regarding best practices for the design, conduct, analysis, interpretation, and reporting of *in vitro*, *in silico*, and *in vivo* nonclinical assays in order to provide reliable nonclinical data for clinical evaluation (Ando *et al.*, 2017; Asakura *et al.*, 2015; Blinova *et al.*, 2017; Kanda *et al.*, 2016). These are covered in an ICH S7B/E14 concept paper that has been endorsed by the ICH General Assembly (Anon., 2018; ICH, 2018).

Current ICH activities are directed at reducing the number of TQT studies to streamline drug development, improving regulatory decision making, and revising product labeling so it accurately reflects risks to both patients and clinicians (Anon., 2020; ICH, 2020; Strauss *et al.*, 2021; Vargas *et al.*, 2021). The concept paper proposes a 2-stage strategy that will include additional approaches validated under S7B to produce better data for the ICH E14 guidance document. This will help regulatory officials create a more efficient, comprehensive, and standardized way to evaluate drug candidates.

The objective of the first stage of the proposed harmonization work is to provide clarity on how to standardize currently developing methods (ie, best practices), including multi-ion channel assays, *in silico* models, *in vitro* human primary assays, hiPSC-CM assays, and *in vivo* evaluations, and how to use this data to support regulatory decision, inform clinical predictions and subsequent clinical assessments. In August 2020, the first stage of ICH E14/S7B Q&As reached Step 2b of the ICH process (Anon., 2020; ICH, 2020). The document will be revised and finalized following the review and evaluation of public comments. The goal is to create a more customizable, fluid, and informative nonclinical testing strategy (Strauss *et al.*, 2021; Vargas *et al.*, 2021).

## RECOMMENDED BEST PRACTICES

After a review of the individual discordant case studies above and consideration of both the limitations of the HESI/FDA database and the additional data included in the Park *et al.* (2018) database analysis (Supplementary Table 3 in Park *et al.*, 2018), many gaps and inconsistencies have been identified in the data submitted and compiled by the FDA.

Nonclinical models do not absolutely replicate the clinical conditions they are used to predict; however, best practices can be used to improve the nonclinical assay quality and achieve the highest predictive power. (1) Assays must cover a broad and appropriate range of drug concentrations or doses to include, if not exceed, estimated clinical exposures, (2) standardized study conditions should be met, and (3) additional drug information, such as plasma protein binding properties, should be used to complement data analysis and interpretation.

In this article, Table 3 lists many important concepts and principles about both *in vitro* and *in vivo* drug safety studies that may lessen inconsistencies between laboratories in the future. Application of these recommended best practices should enhance the overall quality of data and improve the interpretation and translation of nonclinical to clinical data. They are not limited to QT interval translation *per se* (Hanson *et al.*, 2006; Leishman *et al.*, 2012).

**Table 3.** Important Concepts and Principles for *In Vitro* and *In Vivo* Drug Safety Studies to Enhance the Overall Quality of Data and Improve the Interpretation and Translation of Nonclinical and Clinical Data

*In Vitro* Safety Assays
• Testing should occur over a broad range of concentrations based upon projected/estimated clinical exposures.
• Test concentrations should provide a concentration-response curve. Suggestions include: Approximately 5–6 concentrations for *in vitro* (patch clamp/cellular) studies, Approximately 4–5 concentrations for test systems, including the Langendorff isolated heart or cardiac AP.
• Test-appropriate control/standard/reference drugs at relevant concentrations to demonstrate assay sensitivity *(as requested in the ICHS7A &ICHS7B guidelines)*
• Actual test concentrations are considered the gold standard.
• Standardize assay/test conditions (eg, patch clamp protocols, assay perfusate composition).
• Ensure that the NCE is soluble in assay perfusion solutions; inspect solutions for particulates.
• Account for drugs adhering to glass/plastic as warranted *(as per ICH S7B).*
• Ensure that NCE-mediated effects have reached pseudo steady state. • Account for any run-down effect with vehicle alone (detail the run down correction method).
• Independently test major metabolites if confirmed in absorption, distribution, metabolism, and excretion (ADME) studies, particularly in humans.
• Since potential species/tissue differences in expression/function of ion channels exist, assay readout/response should be standardized as a function of the channel/receptor population.
• Review binding and functional ion channel data for similarities/differences in assays.
• Extended application of an NCE in an assay such as hERG trafficking may be warranted.
***In Vivo* Safety Assays**
• Use an appropriate nonclinical species based on the NCE target, pathway, and pharmacological drug class
• Know the statistical power of the assay (ability to detect ΔQTc = 10 ms)
• Test reference drugs as warranted (ie, moxifloxacin for QTc prolongation)
• Determine reference drug exposure levels to confirm assay sensitivity and/or translatability
• Study design: The pro/con of dose escalation vs parallel vs crossover study design imperative
• Apply and indicate the use of appropriate heart rate correction method(s).
• Testing should occur over a broad range of doses to achieve plasma concentrations based upon projected/estimated clinical exposures.
• Consider repeat vs single dose studies if effect(s) are not driven by *C*_max_.
• Repeat dose studies if steady state is achieved.
• Measure blood/serum/plasma concentrations in test animals.
• Account for differences in animal plasma protein binding vs human plasma protein binding.
• Measure drug metabolites as warranted and include them in the interpretation of any findings.
• Use PKPD modeling to refine exposure-response relationship (ie, to resolve QT hysteresis).
• Clinical signs and hemodynamic changes can confound data and limit interpretation of results.
• Significant changes in animal movement/activity/behavior may introduce artifacts and should be documented (or removed) as warranted.
• Application of other biomarkers (ie, body temperature and electrolytes) may help elucidate a mechanism for effects inconsistent with data from *in vitro*/*ex vivo* assays

Abbreviations: hERG, human ether-a-go-go related gene; QTc, corrected QT.

## CONCLUSIONS

As outlined in the ICH guidelines, it is important to evaluate drug-induced changes in the QT interval with integrated risk assessments that take study and testing methodology into account. The relevance of the QT interval as a sole indicator of risk is questionable because many studies confirm that QT prolongation alone does not necessarily precede the development of TdP arrhythmias. Since drug safety scientists must act in a cautious manner, the development of a drug is likely to be discontinued if there are nonclinical indicators of TdP safety risk, no matter how extensive the validation is for the model used.

This article attempts to evaluate the strengths and the limitations. of the assays used to assess QT intervals. Although gaps exist in the translation of nonclinical assays to the clinic, we hope the content of this article allows researchers and clinicians to have increased confidence in those preclinical TdP liability tests and encourages them to develop novel assay platforms and improve current methodologies. If these efforts are successful, they may change regulatory policy; but until the knowledge gaps about the current nonclinical models are filled, there will be limited change to currently used drug safety methods. 

## DECLARATION OF CONFLICTING INTERESTS

The authors declared no potential conflicts of interest with respect to the research, authorship, and/or publication of this article. The following authors are employed in the pharmaceutical industry (at the time of article submission): C.O-R., M.S., M.P., H.M.V., T.W., and J.-P.V. The opinions presented here are those of the authors. No official support or endorsement by the US Food and Drug Administration is intended or should be inferred.

## Notes

**Disclaimer:** The opinions presented here are those of the authors. No official support or endorsement by the U.S. FDA or participating companies is intended or should be inferred.
